# Supplementary material for: Programmable epigenome editing by transient delivery of CRISPR epigenome editor ribonucleoproteins
Source: Nat Commun. 2025 Aug 26;16:7948. doi: 10.1038/s41467-025-63167-x (PMC12381050; doi:10.1038/s41467-025-63167-x)
Supplement: Supplementary file 1 — Supplementary Information [file 41467_2025_63167_MOESM1_ESM.pdf]

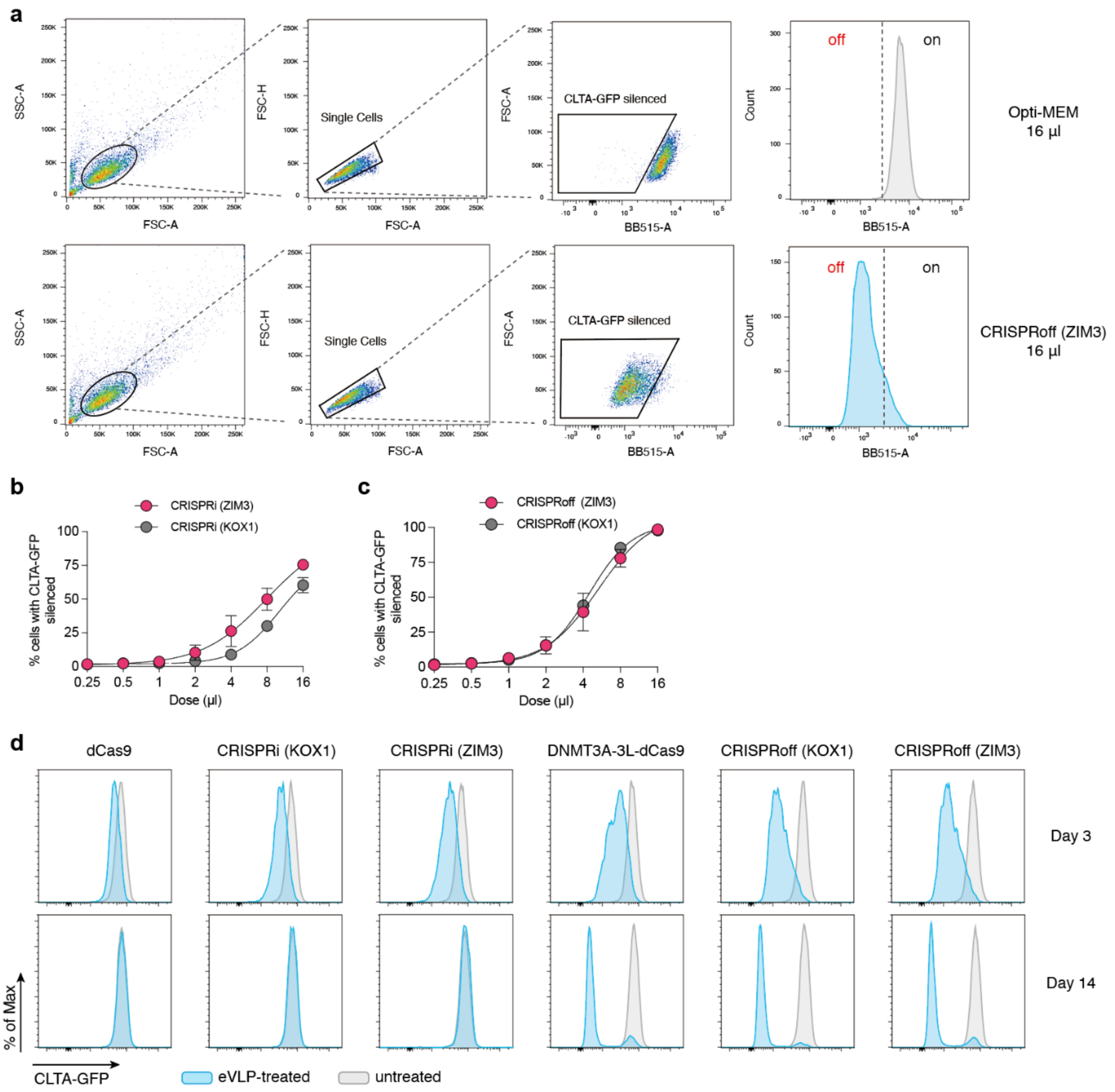

Supplementary Figure 1. Target gene silencing in HEK293T cells by eVLP delivery of different epigenome editors. a, Representative flow cytometry plots showing the gating strategy for quantifying target gene silencing in CLTA-GFP HEK293T reporter cells. In this experiment, Opti-MEM (media without eVLPs) was used as untreated control. **b,** Dose-response curve of CLTA-GFP silencing in HEK293T cells 5 days post-treatment with CRISPRi-eVLP with the ZIM3 or KOX1 KRAB domain. Data were fit to four-parameter logistic curves using nonlinear regression. **c,** Dose-response curve of CLTA-GFP silencing in HEK293T cells 5 days post-treatment with CRISPRoff-eVLP with the ZIM3 or KOX1 KRAB domain. Data were fit to four-parameter logistic curves using nonlinear regression. In **b, c**, Values and error bars reflect mean ± SD of n = 3 biological replicates. **d,** Representative histograms of CLTA-GFP expression in HEK293T cells at day 3 and 14 post-treatment, each treated with the indicated epigenome editor eVLP. Source data are provided as a Source Data file.

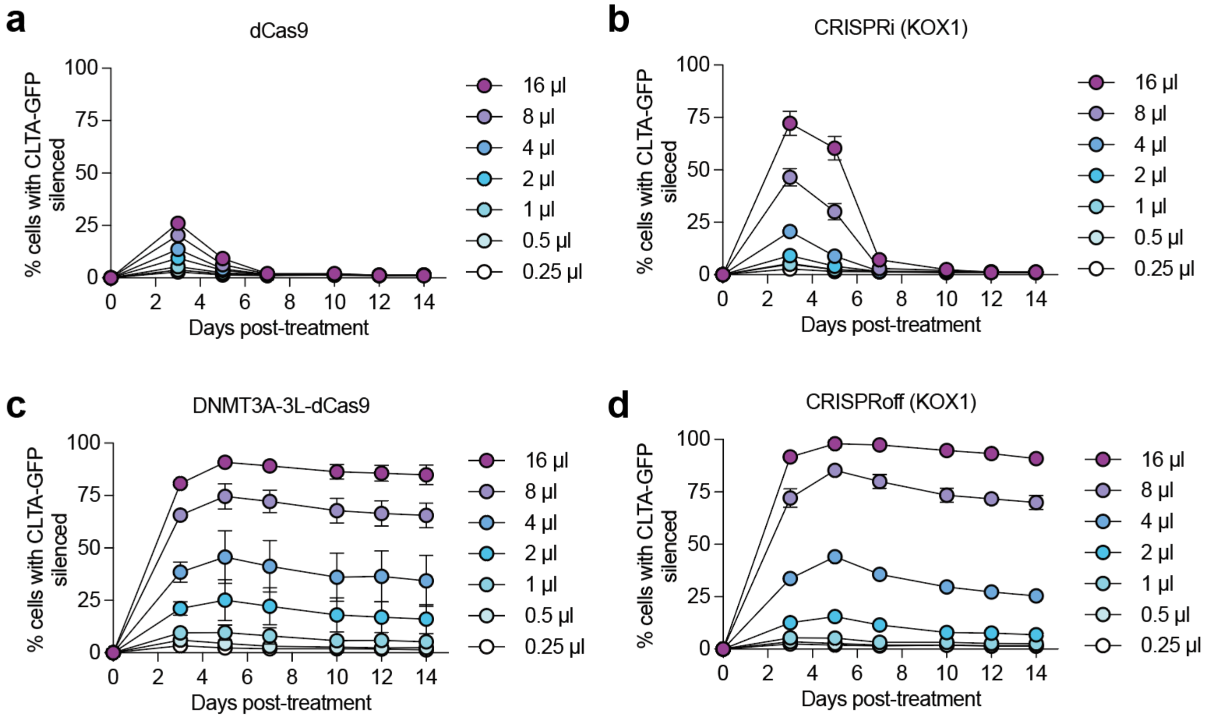

Supplementary Figure 2. Time course of target gene silencing in HEK293T cells by different RENDER delivery of epigenome editors. a, Time course of CLTA-GFP silencing in HEK293T cells post-treatment with different doses of RENDER-dCas9. **b,** Time course of CLTA-GFP silencing in HEK293T cells post-treatment with different doses of RENDER-CRISPRi (KOX1). **c,** Time course of CLTA-GFP silencing in HEK293T cells post-treatment with different doses of RENDER-DNMT3A-3L-dCas9. **d,** Time course of CLTA-GFP silencing in HEK293T cells post-treatment with different doses of RENDER-CRISPRoff (KOX1). In **a-d**, Values and error bars reflect mean ± SD of n = 3 biological replicates. Source data are provided as a Source Data file.

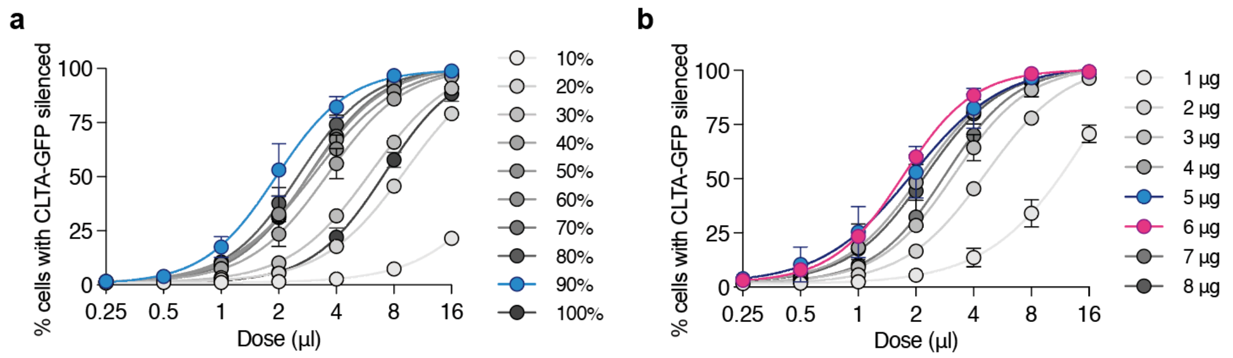

Supplementary Figure 3. Optimization of RENDER-CRISPRoff composition. a, Dose-response curve of CLTA-GFP silencing in HEK293T cells 5 days post-treatment with RENDER-CRISPRoff with varying gag-CRISPRoff to gag-pol plasmid ratios. Label indicates % gag-CRISPRoff plasmid of total amount of gag-CRISPRoff and gag-pol plasmids. Blue indicates v2. Data were fit to four-parameter logistic curves using nonlinear regression. **b,** Dose-response curve of CLTA-GFP silencing in HEK293T cells 5 days post-treatment with RENDER-CRISPRoff with varying amounts of sgRNA expressing plasmid. Blue indicates v2, magenta indicates v3. Data were fit to four-parameter logistic curves using nonlinear regression. In **a, b**, Values and error bars reflect mean ± SD of n = 3 biological replicates. Source data are provided as a Source Data file.

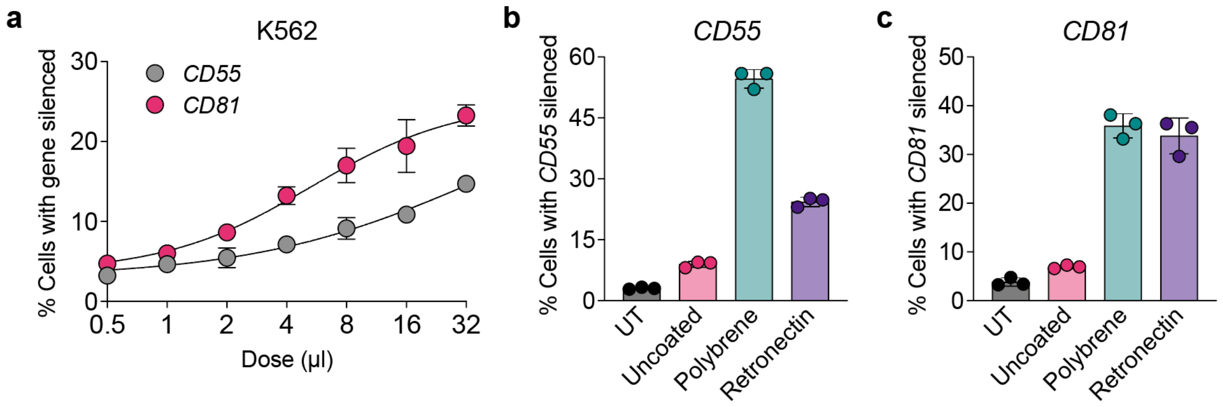

Supplementary Figure 4. Optimization of RENDER-CRISPRoff transduction in K562 cells. **a,** Dose-response curves of *CD55* silencing and *CD81* silencing in K562 cells 7 days post-treatment with different doses of RENDER-CRISPRoff v3. Values and error bars reflect mean ± SD of n = 3 biological replicates. Data were fit to four-parameter logistic curves using nonlinear regression. **b, c,** Quantification of (**b**) *CD55* and (**c**) *CD81* silencing in K562 cells 7 days post-treatment with RENDER-CRISPRoff v3 (10 µl), comparing uncoated (uncoated), polybrene- and RetroNectin-coated plates. UT, untreated. In **b, c**, Data are shown as individual data points and mean ± SD for n = 3 biological replicates. Source data are provided as a Source Data file.

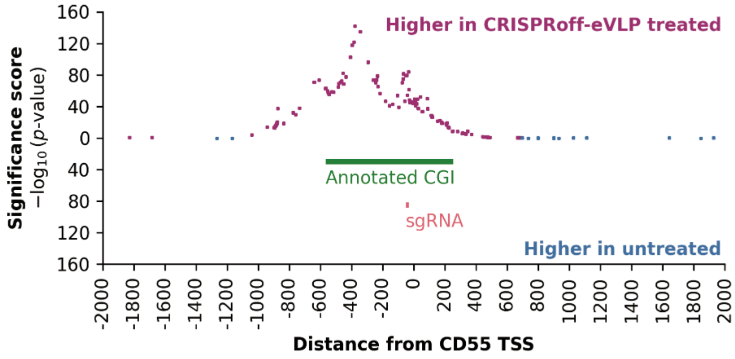

Supplementary Figure 5. DNA methylation profile of RENDER-CRISPRoff *CD55*-sgRNA treated Jurkat cells. Zoomed-in Manhattan plot displaying differentially methylated CpGs across a ±2 kilobase pairs window from the *CD55* transcription start site (TSS) between Jurkat cells treated with RENDER-CRISPRoff v3 targeting *CD55* and untreated cells (16 days post-treatment) analyzed by WGEMseq. Magenta dots represent CpGs that are more methylated in RENDER-CRISPRoff-treated cells and blue dots represent CpGs that are more methylated in untreated cells. Annotated CpG island (CGI) is shown in green and *CD55* targeting sgRNA in red.

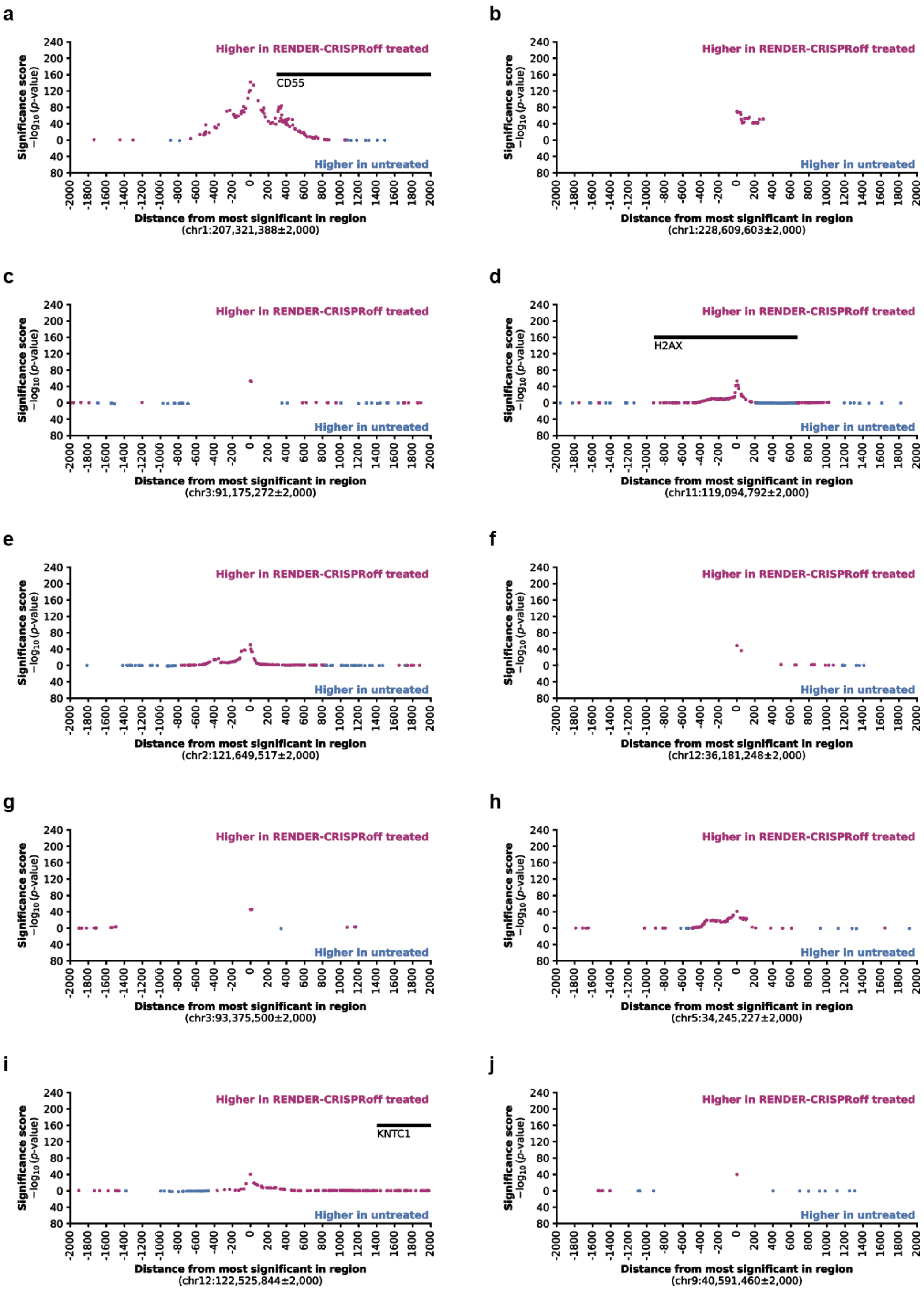

Supplementary Figure 6. Differentially more methylated regions in RENDER-CRISPRoff treated Jurkat cells. Top ten most significant regions showing increased DNA methylation in RENDER-CRISPRoff treated versus untreated Jurkat cells, as analyzed by whole-genome EM-seq. Magenta dots indicate CpG sites with increased methylation in RENDER-CRISPRoff-treated cells, whereas blue dots indicate CpG sites with increased methylation in untreated cells. Black bars represent annotated genes.

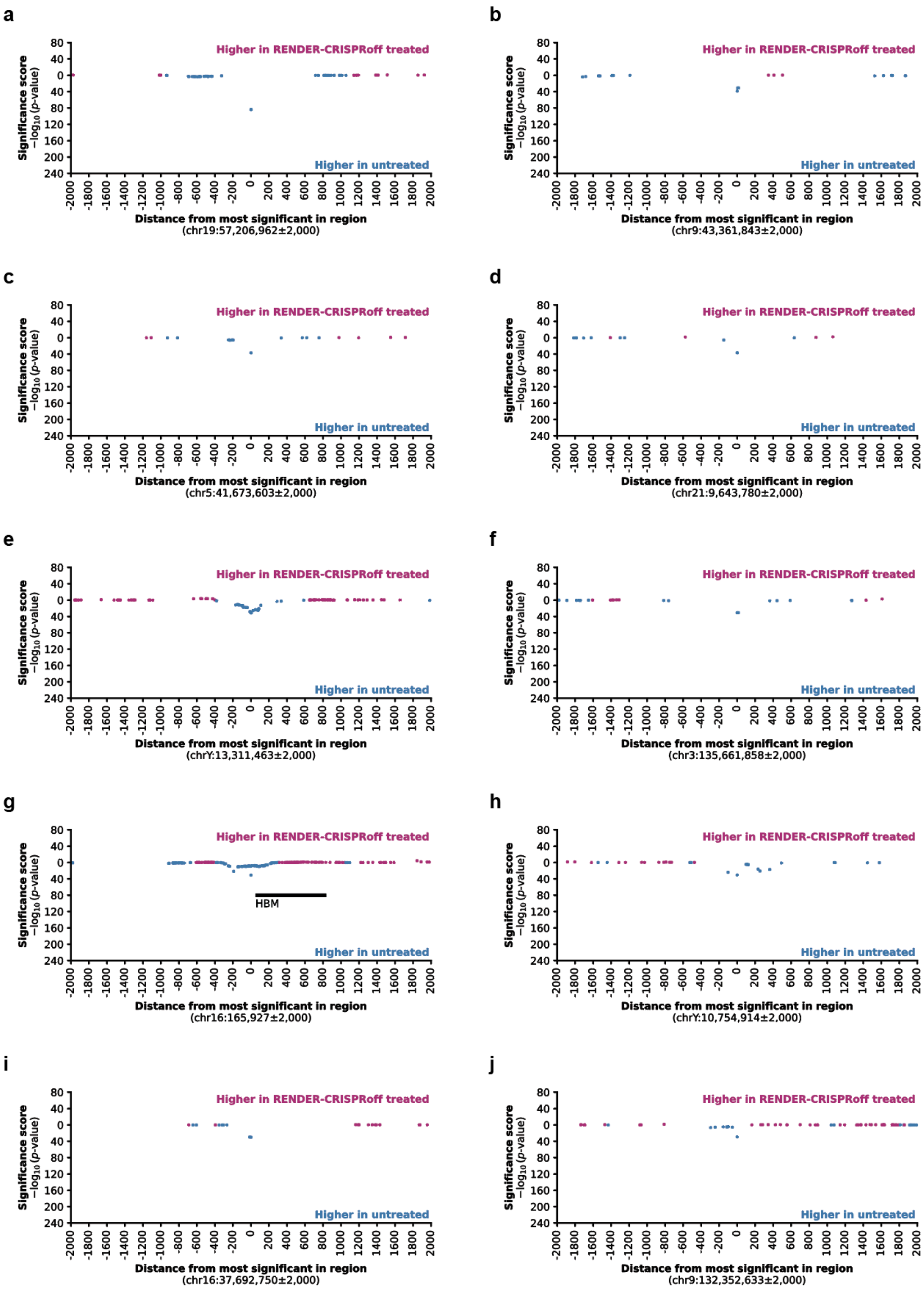

Supplementary Figure 7. Differentially less methylated regions in RENDER-CRISPRoff treated Jurkat cells. Top ten most significant regions showing reduced DNA methylation in RENDER-CRISPRoff treated versus untreated Jurkat cells, as analyzed by whole-genome EM-seq. Magenta dots represent CpGs with increased methylation in treated cells, while blue dots represent CpGs with increased methylation in untreated cells. Black bars represent annotated genes.

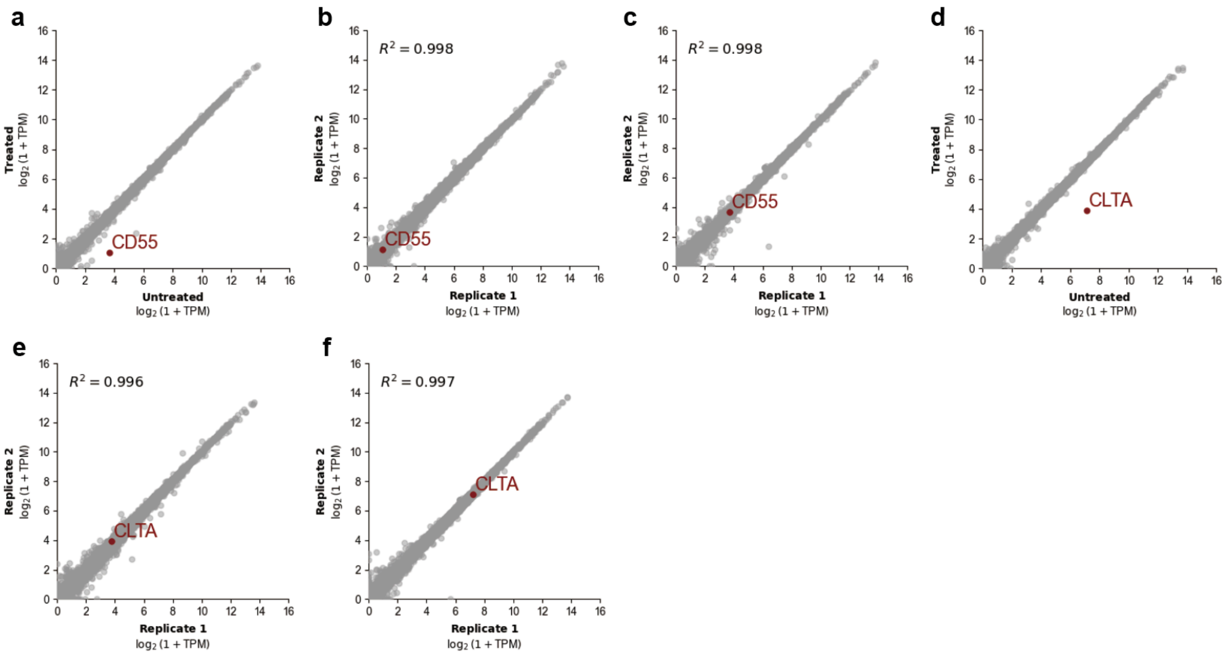

Supplementary Figure 8. RNA-seq analysis of RENDER-CRISPRoff treated Jurkat and HEK293T cells. a, RNA-seq plots of Jurkat cells treated with RENDER-CRISPRoff v3 packaged with sgRNAs targeting *CD55* compared to untreated Jurkat cells. The data are representative of the average of two independent replicates. **b, c,** Comparison between two replicates of RNA-seq of (**b**) Jurkat cells treated with RENDER-CRISPRoff v3 packaged with *CD55*-sgRNAs, and (**c**) untreated Jurkat cells. **d,** RNA-seq plots of HEK293T cells treated with RENDER-CRISPRoff v3 packaged with sgRNAs targeting *CLTA* compared to untreated HEK293T cells. The data are representative of the average of two independent replicates. **e, f,** Comparison between two replicates of RNA-seq of (**e**) Jurkat cells treated with RENDER-CRISPRoff v3 packaged with *CLTA*-sgRNAs, and (**f**) untreated HEK293T cells.

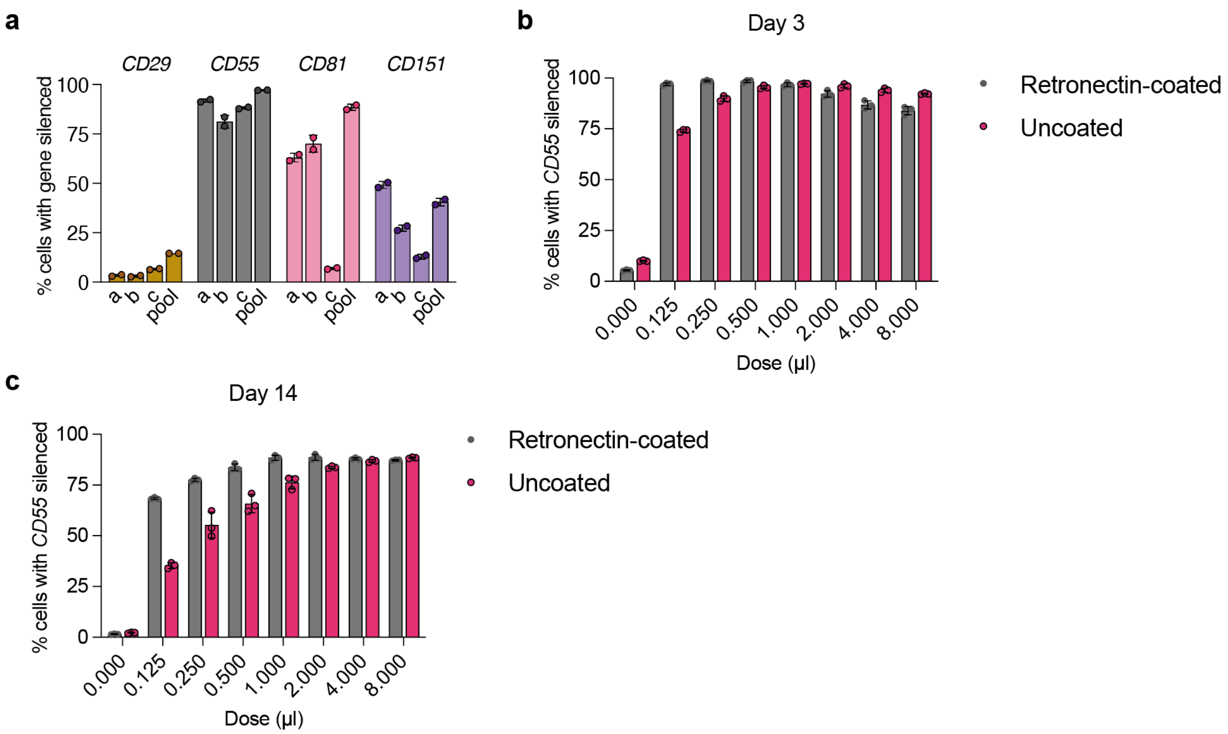

Supplementary Figure 9. Optimization of RENDER-CRISPRoff transduction in Jurkat cells. **a,** Quantification of *CD29*, *CD55*, *CD81* and *CD151* silencing in Jurkat cells 14 days post treatment with RENDER-CRISPRoff v3 packaged with individual sgRNAs (a,b and c) or a pool of three sgRNAs (a, b, c). Data are shown as individual data points and mean ± SD for n = 2 biological replicates. **b,** Quantification of *CD55* silencing in Jurkat cells 3 days post treatment with different doses of RENDER-CRISPRoff v3, comparing RetroNectin-coated and uncoated plates for transduction. **c,** Quantification of *CD55* silencing in Jurkat cells 14 days post treatment with different doses of RENDER-CRISPRoff v3, comparing RetroNectin-coated and uncoated plates for transduction. In **b, c**, Data are shown as individual data points and mean ± SD for n = 3 biological replicates. Source data are provided as a Source Data file.

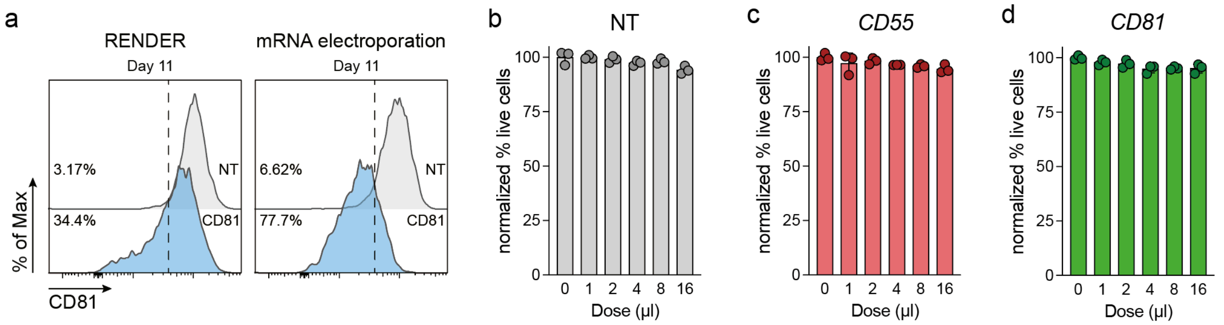

Supplementary Figure 10. RENDER-CRISPRoff treatment in primary human T cells. a, Representative histogram plot of *CD81* expression on T cells 11 days post-treatment with RENDER-CRISPRoff v3 (5 µl) targeting *CD81* (blue) or non-targeting (gray), or post-electroporation with CRISPRoff-mRNA and *CD81* targeting (blue) or non-targeting (gray) sgRNAs. Percentages denote % cells with *CD81* silenced. **b-d,** Quantification of live T cells 2 days post RENDER-CRISPRoff v3 treatment, with (**b**) non-targeting (NT) sgRNAs, (**c**) *CD55* targeting sgRNAs, and (**d**) *CD81* targeting sgRNAs. In **b-d**, Data are shown as individual data points and mean ± SD for n = 3 biological replicates. The data were normalized to the untreated control group. Source data are provided as a Source Data file.

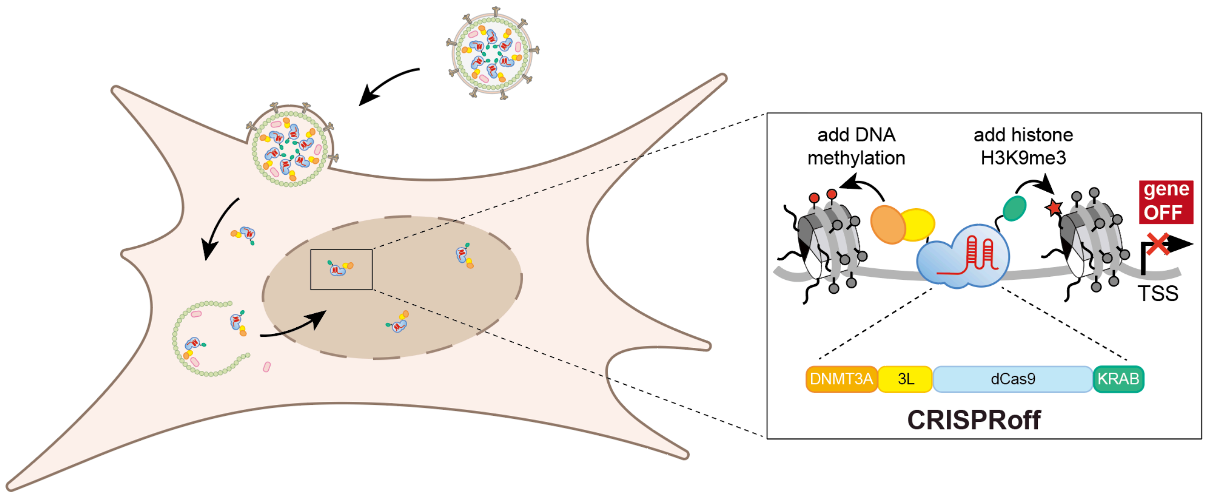

Supplementary Figure 11. Schematic of RENDER-CRISPRoff delivery and epigenome editing. The envelope glycoprotein protein on the VLP surface facilitates fusion between the VLP and host cell membranes. Within the cytoplasm, the mature core packaging editor RNPs dissociate, and RNPs are released. The nuclear localization signal on the editor proteins guides the RNPs into the nucleus. Once in the nucleus, the RNPs recognize and bind to the target DNA sequence, leading to the deposition of DNA methylation and histone modifications, which ultimately represses target gene expression.
