# Supplementary material for: Programmable epigenome editing by transient delivery of CRISPR epigenome editor ribonucleoproteins
Source: Nat Commun. 2025 Aug 26;16:7948. doi: 10.1038/s41467-025-63167-x (PMC12381050; doi:10.1038/s41467-025-63167-x)
Supplement: Supplementary file 2 — Description of Additional Supplementary Information [file 41467_2025_63167_MOESM2_ESM.pdf]

Description of Additional Supplementary Files

File Name: Supplementary Data 1
Description: Recombinant DNA.

File Name: Supplementary Data 2
Description: Protospacer sequences.

File Name: Supplementary Data 3
Description: Flow cytometry antibodies and dyes.
