# Supplementary material for: Programmable epigenome editing by transient delivery of CRISPR epigenome editor ribonucleoproteins
Source: Nat Commun. 2025 Aug 26;16:7948. doi: 10.1038/s41467-025-63167-x (PMC12381050; doi:10.1038/s41467-025-63167-x)
Supplement: Supplementary file 6 — Reporting Summary [file 41467_2025_63167_MOESM6_ESM.pdf]

nature portfolio

Corresponding author(s): James Karlo Nuñez

Last updated by author(s): Jul 11, 2025

Reporting Summary

Nature Portfolio wishes to improve the reproducibility of the work that we publish. This form provides structure for consistency and transparency in reporting. For further information on Nature Portfolio policies, see our Editorial Policies and the Editorial Policy Checklist.

Statistics

For all statistical analyses, confirm that the following items are present in the figure legend, table legend, main text, or Methods section.

n/a	Confirmed	
[ ]	[x]	The exact sample size (*n*) for each experimental group/condition, given as a discrete number and unit of measurement
[ ]	[x]	A statement on whether measurements were taken from distinct samples or whether the same sample was measured repeatedly
[ ]	[x]	The statistical test(s) used AND whether they are one- or two-sided<br>*Only common tests should be described solely by name; describe more complex techniques in the Methods section.*
[x]	[ ]	A description of all covariates tested
[x]	[ ]	A description of any assumptions or corrections, such as tests of normality and adjustment for multiple comparisons
[ ]	[x]	A full description of the statistical parameters including central tendency (e.g. means) or other basic estimates (e.g. regression coefficient) AND variation (e.g. standard deviation) or associated estimates of uncertainty (e.g. confidence intervals)
[x]	[ ]	For null hypothesis testing, the test statistic (e.g. *F*, *t*, *r*) with confidence intervals, effect sizes, degrees of freedom and *P* value noted<br>*Give P values as exact values whenever suitable.*
[x]	[ ]	For Bayesian analysis, information on the choice of priors and Markov chain Monte Carlo settings
[x]	[ ]	For hierarchical and complex designs, identification of the appropriate level for tests and full reporting of outcomes
[x]	[ ]	Estimates of effect sizes (e.g. Cohen's *d*, Pearson's *r*), indicating how they were calculated

Our web collection on statistics for biologists contains articles on many of the points above.

Software and code

Policy information about availability of computer code

Data collection	BD FACSDiva was used to collect flow cytometry data.
Data analysis	Frequency, mean, and standard deviations were calculated using GraphPad Prism 10.

For manuscripts utilizing custom algorithms or software that are central to the research but not yet described in published literature, software must be made available to editors and reviewers. We strongly encourage code deposition in a community repository (e.g. GitHub). See the Nature Portfolio guidelines for submitting code & software for further information.

Data

Policy information about availability of data

All manuscripts must include a data availability statement. This statement should provide the following information, where applicable:

- Accession codes, unique identifiers, or web links for publicly available datasets
- A description of any restrictions on data availability
- For clinical datasets or third party data, please ensure that the statement adheres to our policy

WGEM-seq and RNA-seq sequencing data files are available from NCBI SRA under accession code PRJNA1195923.

Research involving human participants, their data, or biological material

Policy information about studies with human participants or human data. See also policy information about sex, gender (identity/presentation), and sexual orientation and race, ethnicity and racism.

Reporting on sex and gender	This study does not involve human research participants.
Reporting on race, ethnicity, or other socially relevant groupings	N/A
Population characteristics	N/A
Recruitment	N/A
Ethics oversight	N/A

Note that full information on the approval of the study protocol must also be provided in the manuscript.

Field-specific reporting

Please select the one below that is the best fit for your research. If you are not sure, read the appropriate sections before making your selection.

☒ Life sciences ☐ Behavioural & social sciences ☐ Ecological, evolutionary & environmental sciences

For a reference copy of the document with all sections, see nature.com/documents/nr-reporting-summary-flat.pdf

Life sciences study design

All studies must disclose on these points even when the disclosure is negative.

Sample size	Sample sizes were determined based on preliminary experiments and/or experimental design used in previous related studies.
Data exclusions	Two samples were excluded in Fig. 4c due to failing staining.
Replication	All experiments were repeated more than once. All attempts were successful.
Randomization	All control and tested conditions were assigned randomly.
Blinding	Blinding was not relevant to the experiments due to the experimental settings described in methods and figure legends.

Reporting for specific materials, systems and methods

We require information from authors about some types of materials, experimental systems and methods used in many studies. Here, indicate whether each material, system or method listed is relevant to your study. If you are not sure if a list item applies to your research, read the appropriate section before selecting a response.

Materials & experimental systems

n/a	Involved in the study	
☐	☒	Antibodies
☐	☒	Eukaryotic cell lines
☒	☐	Palaeontology and archaeology
☒	☐	Animals and other organisms
☒	☐	Clinical data
☒	☐	Dual use research of concern
☒	☐	Plants

Methods

n/a	Involved in the study	
☒	☐	ChIP-seq
☐	☒	Flow cytometry
☒	☐	MRI-based neuroimaging

Antibodies

Antibodies used	Mouse-anti FLAG antibody (Sigma-Aldrich, F3165); Goat anti-mouse secondary antibody (LI-COR, IRDye 680RD); APC mouse-anti human CD29 antibody (Biolegend, #303008); APC mouse-anti human CD55 antibody (Biolegend, #311312); FITC mouse-anti human CD55 antibody (Biolegend, #311306); PE mouse-anti human CD81 antibody (Biolegend, #349506); Pacific Blue mouse-anti human CD81 antibody (Biolegend, #349516); PE mouse-anti human CD151 antibody (Biolegend, #350408); Mouse-anti human Tau antibody (Santa Cruz Biotechnology, sc-21796); Goat-anti mouse IgG Alexa Fluor® 488 antibody (abcam, ab-150117).

Validation	All commercial antibodies were validated by the manufacturers for western blot or flow cytometry.

Eukaryotic cell lines

Policy information about cell lines and Sex and Gender in Research

Cell line source(s)	HEK293T, Lenti-X, Jurkat, HeLa, h-TERT RPE-1, HepG2, U87 and SH-SY5Y cells were obtained from the UC Berkeley Cell Culture Facility. Human peripheral blood mononuclear cells were obtained from STEMCELL.
Authentication	Authentication of HEK293T, Lenti-X, Jurkat, HeLa, h-TERT RPE-1, HepG2, U87 and SH-SY5Y cells was performed using STR by the UC Berkeley Cell Culture Facility. Human peripheral blood mononuclear cells were authenticated by the supplier using STR.
Mycoplasma contamination	All cell lines were tested negative for mycoplasma.
Commonly misidentified lines (See ICLAC register)	None used.

Plants

Seed stocks	N/A
Novel plant genotypes	N/A
Authentication	N/A

Flow Cytometry

Plots

Confirm that:

- [ ] The axis labels state the marker and fluorochrome used (e.g. CD4-FITC).
- [ ] The axis scales are clearly visible. Include numbers along axes only for bottom left plot of group (a 'group' is an analysis of identical markers).
- [x] All plots are contour plots with outliers or pseudocolor plots.
- [x] A numerical value for number of cells or percentage (with statistics) is provided.

Methodology

Sample preparation	Samples were prepared as described in Methods.
Instrument	BD FACSymphony A1 Cell Analyzer and Attune NxT (Thermo Fisher Scientific) were used.
Software	FlowJo v10 10.10 was used.
Cell population abundance	Gated cell population abundance is presented on the original flow plots or in summary graphs.
Gating strategy	Gating strategy is shown in Extended Data Fig.1.

- [x] Tick this box to confirm that a figure exemplifying the gating strategy is provided in the Supplementary Information.
